# Implications of ADAM17 activation for hyperglycaemia, obesity and type 2 diabetes

**DOI:** 10.1042/BSR20210029

**Published:** 2021-05-14

**Authors:** Jennifer Matthews, Sofia Villescas, Lakshini Herat, Markus Schlaich, Vance Matthews

**Affiliations:** 1 Dobney Hypertension Centre, School of Biomedical Science - Royal Perth Hospital Unit, University of Western Australia, Crawley, WA 6009, Australia; 2 Dobney Hypertension Centre, School of Medicine – Royal Perth Hospital Unit, University of Western Australia, Crawley, WA 6009, Australia; 3 Department of Cardiology and Department of Nephrology, Royal Perth Hospital, Perth, WA 6000, Australia

**Keywords:** ADAM17, hypertension, metabolic syndromes, obesity, TACE, type 2 diabetes

## Abstract

In this review, we focus specifically on the role that the metalloproteinase, A Disintegrin and Metalloproteinase 17 [ADAM17] plays in the development and progression of the metabolic syndrome. There is a well-recognised link between the ADAM17 substrate tumour necrosis factor α (TNF-α) and obesity, inflammation and diabetes. In addition, knocking out ADAM17 in mice leads to an extremely lean phenotype. Importantly, ADAM17-deficient mice exhibit one of the most pronounced examples of hypermetabolism in rodents to date. It is vital to further understand the mechanistic role that ADAM17 plays in the metabolic syndrome. Such studies will demonstrate that ADAM17 is a valuable therapeutic target to treat obesity and diabetes.

## Introduction

There is increasing evidence showing the link between metabolic syndrome and cardiovascular disease, chronic kidney disease, stroke and diabetes [1]. The metabolic syndrome is defined as a cluster of independent risk factors which coexist, leading to an increased risk of the above-mentioned diseases. Geographically, studies have shown varied levels of the prevalence of the metabolic syndrome, ranging from approximately 35% in the United States [2], 36% in Australia [3], up to 26% in Europe [1] and up to 37% in Asia [1].

There is a slightly different definition of metabolic syndrome between the NCEP ATPIII Criteria [4] and the World Health Organization (WHO) [5]. Although their criteria is very similar in many aspects, there are some slight differentiations based on what they believe to be the predominant causes of metabolic syndrome.

The NCEP [4] categorises an individual having metabolic syndrome when they have at least three out of five of the following markers:

1) Waist circumference > 102 cm (40 inches) in males and >88 cm (35 inches) in females;
2) Elevated triglycerides > 1.7 mmol/l (150mg/dl);
3) Lowered high-density lipoprotein (HDL) cholesterol levels < 1.0 mmol/l (40 mg/dl) in males, <1.3 mmol/l (50 mg/dl) in females;
4) Elevated fasting glucose > 5.6 mmol/l (100 mg/dl) (due to insulin resistance) and/or
5) Elevated blood pressure > 130/85 mmHg.

WHO [4,5] categorises an individual as having metabolic syndrome when they have insulin resistance or diabetes, plus at least two out of five of the following markers:

1) Waist/Hip ratio: >0.90 in males and >0.85 in females or body mass index (BMI) > 30 kg/m^2^;
2) Elevated triglycerides > 1.7 mmol/l (150 mg/dl);
3) Lowered HDL cholesterol levels < 0.9 mmol/l (35 mg/dl) in males, <1 mmol/l (39 mg/dl) in females;
4) Elevated blood pressure > 140/90 mmHg;
5) Microalbuminuria: Urinary albumin excretion rate ≥ 20 μg/min or albumin:creatinine ratio ≥ 30 mg/g.

Some lifestyle factors associated with metabolic syndrome include obesity, lack of physical activity and some genetic factors, such as mutations in genes regulating lipid metabolism [3].

**Figure 1:**
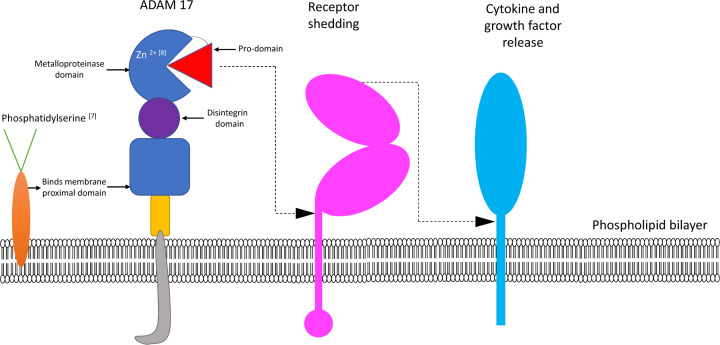
Schematic representation of ADAM17-mediated shedding Phosphatidylserine transported to the outside of the membrane is required for ADAM17 activation [7]. ADAM enzymes are dependent on Zn^2+^ for activation and absence of Zn^2+^ renders the enzymes proteolytically inactive [8]. The function of ADAM17 includes shedding of receptors, growth factors and cytokines.

Obesity is a rapidly growing global pandemic which has almost tripled since 1975. As of 2016, more than 1.9 billion adults alone (>18 years) were overweight and of these, over 600 million were classified as obese [6]. Low-grade, chronic inflammation has been associated with the development of metabolic syndrome, mediated via activation of various cytokines. Here, we focus specifically on the role of the metalloproteinase A Disintegrin and Metalloproteinase 17 [ADAM17] (Figure 1) and how it may impact the metabolic syndrome.

## What is ADAM17?

To date, the mouse genome contains at least 34 *Adam* genes, while the human genome contains at least 27 *Adam* loci [9,10]. ADAM17 (also known as TACE or tumour necrosis factor α (TNF-α) converting enzyme), was first discovered by Black et al. in 1997 [11,12]. ADAM17 consists of numerous domains which include the pro-domain, metalloproteinase domain, disintegrin domain and membrane proximal domain. The pro-domain ensures that ADAM17 remains in an inactive state, until it is cleaved which thereby allows the metalloproteinase domain to become catalytically active [13]. Zinc binding to the metalloproteinase domain is also required for activity [8,14]. Interestingly, the disintegrin domain also possesses adhesive properties [14]. ADAM17 is a metalloproteinase that has been identified as the sheddase for a broad range of membrane-bound proteins expressed on numerous cell types including haematopoietic cells [15–17]. It plays a major role in chemokine/cytokine shedding, cell signalling, proliferation and growth [18]. As you will discover in this review, ADAM17 has both beneficial and detrimental effects. Although it has been shown to be beneficial for embryonic development, liver health and adipocyte differentiation, it is also implicated in the pathogenesis of many different diseases including, but not limited to cancer [19], heart disease [20], diabetes [21,22], rheumatoid arthritis [23] and Alzheimer’s disease [24]. In this review, we aim to highlight the impact of ADAM17 on the progression of Metabolic Syndrome.

## Functional importance of ADAM17

Proteolysis usually occurs at the membrane-adjacent part of the substrate, many of which are receptors [25]. If the receptor shedding occurs before the ligand binding, the solubilised receptor can inhibit the ligand binding to cell surface receptors. This will be discussed in more detail later.

Although there are marked detrimental consequences of having excess amounts of ADAM17 activity, there is also a need for a balanced discussion on the important roles that it plays too.

### Embryonic development

ADAM17 is particularly important for embryonic development as studies have shown that the embryos of ADAM17-deficient mice have defects in the mammary epithelium, vascular system, lung, eye, hair, heart and skin and therefore may die early on in pregnancy or even a few days after birth. Those mice that survived have reduced lymphocyte numbers, impaired T- and B-cell development and reduced body weight [8,26].

### Liver health

The impact that ADAM17 has on liver health is controversial and it can act like ‘Jekyll and Hyde’ by playing both beneficial and detrimental roles in liver biology. Studies involving up-regulation of this metalloproteinase have been instrumental in highlighting that ADAM17 plays a major role in hepatosteatosis and liver inflammation, ultimately contributing to the development of metabolic syndrome [27]. However, there is also research indicating that ADAM17 plays a role in protecting hepatocytes from apoptosis in cases of drug-induced liver failure and that adenoviral delivery of ADAM17 prevented acetaminophen induced liver failure in a clinically relevant model of Fas-dependent fulminant hepatitis [28].

### Adipocyte differentiation

ADAM17 may sometimes act like a ‘double-edged sword’ in relation to adipocyte differentiation. Although there are many studies demonstrating the detrimental effect of ADAM17 shedding on adiposity, one of its substrates, pre-adipocyte factor 1 (Pref-1) may be beneficial as discussed in more detail below. Interestingly, Pref-1 inhibits adipocyte differentiation [29].

## Implications of ADAM17 in the metabolic syndrome

### Obesity

ADAM17 was first identified as being responsible for shedding of the pro-inflammatory cytokine TNF-α [12]. There is a well-recognised link between TNF-α and obesity, inflammation and diabetes and an increased expression of TNF-α is found in the adipose tissue of obese and insulin-resistant animal and human models [30]. The TNF-α in human adipose tissue positively correlates with BMI, percentage of body fat and hyperinsulinaemia and studies have shown that weight loss decreases TNF-α levels [30].

Knocking out ADAM17 in mice leads to extremely lean animals. ADAM17-deficient mice exhibit one of the most pronounced examples of hypermetabolism reported in a rodent system to date. Elevated levels of uncoupling protein-1 in the brown adipose tissue of ADAM17-deficient mice compared with wildtype mice suggests that this lowered ADAM17 activity is linked to increased sympathetic outflow [31]. Interestingly, we have recently shown that sympathoexcitation in white adipose tissue is associated with beiging of adipose tissue [32].

In an independent study [33], high-fat diet (HFD) treated TaceMx1 mice (which have ADAM17 knocked out in haematopoietic cells) were found to have lower adipose tissue weights, systolic blood pressure, fasting glucose, fasting lipid levels and serum adiponectin levels. In addition, ADAM17 inactivation increased energy expenditure and oxidation of both fat and carbohydrate and improved glucose tolerance and insulin sensitivity when compared with the HFD wildtype (WT) mice.

### Diabetes/insulin resistance

As mentioned previously, ADAM17 expression is significantly increased in the liver and adipose tissue of mice that have been fed HFD and it is positively associated with the development of insulin resistance and hepatosteatosis [33]. As increased ADAM17 expression is correlated with insulin resistance [30], it is likely that decreasing ADAM17 activity via various therapeutic strategies may increase insulin sensitivity and ultimately have a beneficial effect on obesity.

One study has highlighted that when ADAM17 is activated within the white adipocytes, it leads to the expression of inflammatory molecules such as Interleukin 6 (IL-6), Monocyte Chemotactic Protein 1 (MCP-1) and Suppressor of Cytokine Signalling 3 (SOCS3) [30,34]. This expression then leads to a low-grade inflammatory state that forces the macrophages to migrate into adipose tissue where they mediate enhanced insulin resistance [30].

### Hypertension

Neurogenic hypertension is a form of high blood pressure which eventuates due to hyperactivation of the sympathetic nervous system. One mechanism by which neurogenic hypertension may occur is by ADAM17 mediated Angiotensin-converting enzyme type 2 (ACE2) shedding which results in loss of membrane-bound ACE2. This may promote high blood pressure as a consequence of a failure of ACE2 to convert angiotensin-II (vasoconstrictor) into angiotensin 1-7 (vasodilator) [35,36]. These findings are supported by studies highlighting that ADAM17 activation on glutamatergic neurons has been demonstrated to result in sympathoexcitation which may induce neurogenic hypertension [37].

## Substrates for ADAM17 and implications for obesity and type 2 diabetes

**Figure 2:**
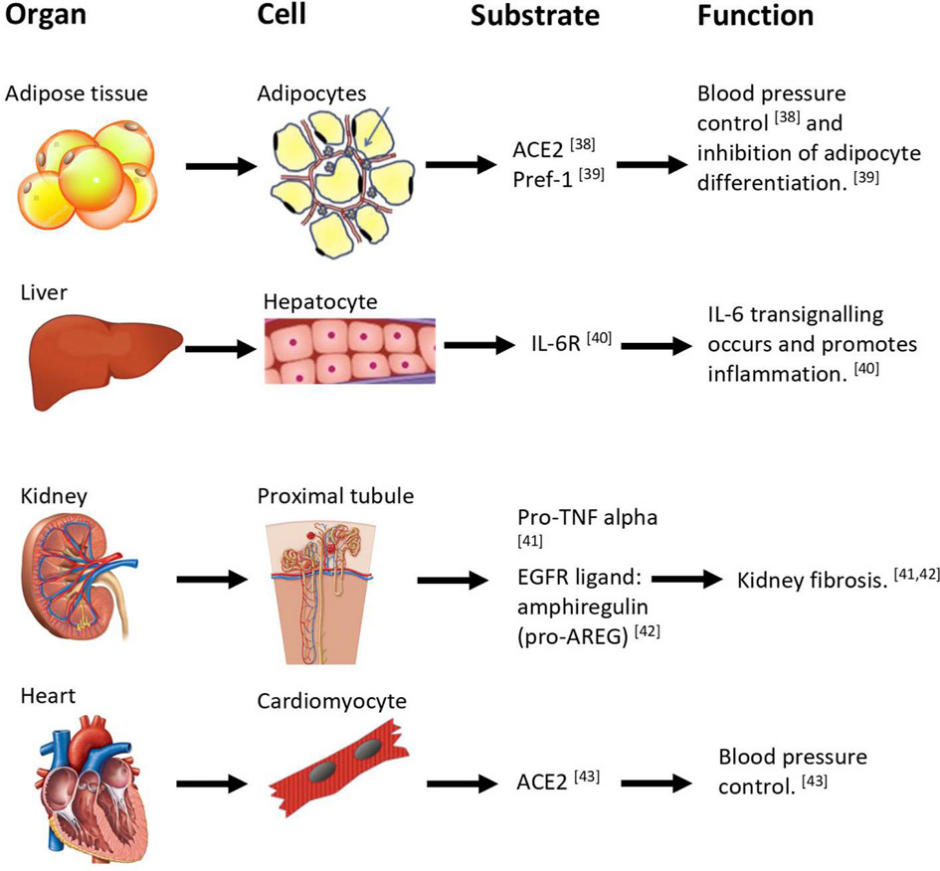
The effect of various ADAM17 substrates on bodily organs and cell tissue function ADAM17 shedding causes an alteration to homeostatic cell function due to the profound effects of ADAM17-mediated cleaved proteins [38–43] which circulate systemically and act at the molecular and cellular levels.

Since the discovery of ADAM17, a vast array of proteins have been shown to be targets for shedding by this protease (Figure 2). It has been shown that the high glucose levels that exist during diabetes may be mediating increased expression of ADAM17 in cell types such as the mesangial cells [44] and therefore result in the subsequent cleavage of substrates. ADAM17 has also been shown to be governed by a number of cytokines. For instance IL-1β and TNFα may increase ADAM17 expression [45]. Additionally, external factors such as hypoxia have been shown to induce ADAM17 expression within human glioma cells by promoting Sp1 mediated transcription of the *Adam17* gene [46]. The shedding event often promotes biological processes that may influence the metabolic syndrome. We will now discuss an important selection of ADAM17 substrates.

### TNF-α

TNF-α was the first white adipose tissue derived inflammatory cytokine that was recognised to confer a link amongst obesity, inflammation and diabetes. It appears to be a crucial contributor to adipokine dysregulation in adipocytes [12,30]. Interestingly, ADAM17-mediated cleavage of TNF-α is implicated in both central and peripheral inflammation [18].

### TNF-R1 and TNF-R2

The receptors for the cytokine TNF-α are TNF-R1 and TNF-R2. Both are shed by ADAM17 to release a soluble receptor which is between 30 and 40 kDa [47,48]. These soluble receptors act to inhibit the binding of circulating TNF-α to the membrane-bound TNF receptors [49,50].

### Pre-adipocyte factor 1

As mentioned previously, ADAM17 may play a beneficial role in reducing adiposity as it is responsible for releasing Pref-1, which is known to inhibit adipose tissue differentiation. Pref-1 belongs to a ‘family of epidermal growth factor-like repeat containing proteins’ which are highly expressed in 3T3-L1 cells and it is reduced during adipocyte differentiation.

Although it is synthesised as a transmembrane protein, Pref-1 is processed to generate both a large 50-kDa soluble form, as well as small soluble forms. However, it is important to note that only the large soluble form is biologically active and inhibits adipogenesis. It is ADAM17 which releases this large 50-kDa soluble form. Mice lacking Pref-1 show accelerated fat deposition, but those mice that have overexpression of Pref-1 show reduced expression of adipocyte markers, as well as a decrease in fat mass [29].

### IL-6R

IL-6 is a key regulator of a multitude of immune responses which range from bacterial infections to liver regeneration. ADAM17 is one of the metalloproteinases that mediates the release of the IL-6R from the cell membrane [51]. We and others have shown that when the 80-kDa membrane-bound form of the receptor is subjected to shedding by ADAM17, a 60-kDa agonistic soluble IL-6R (sIL-6R) is generated [52]. The process of sIL-6R binding to circulating IL-6 and then subsequently stimulating cells of the body is called trans-signalling [52]. Trans-signalling has been linked to cancer and we have also shown that trans-signalling promotes obesity-induced adipose tissue inflammation [53].

### Epiderminal growth factor receptor ligands (in particular epiregulin)

A feedback signalling cascade known as the epiderminal growth factor receptor ligands (EGFR)/ADAM17 axis occurs through the shedding of the EGFR ligands by ADAM17. This cascade is particularly sensitive to external triggers such as cigarette smoke and bacterial toxins, resulting in increased shedding of many growth factors, cytokines and cytokine receptors which are all substrates of ADAM17 [54]. ADAM17 has been implicated in the shedding of the EGFR ligands TGF-α and HB-EGF. The other EGFR ligands amphiregulin and epiregulin have been identified as novel substrates of ADAM17 [55]. It was found that eNOS^−/−^ db/db mice with advanced Diabetic Neuropathy who were treated with the EGFR inhibitor Erlotinib displayed decreases in fasting blood glucose levels, improved glucose tolerance and insulin sensitivity and lowered levels of adiponectin [56].

### Fractalkine

Fractalkine (FKN) is the sole member of the CX3C chemokine family and is a potent chemoattractor for T cells, monocytes and natural killer cells. Although FKN is predominantly expressed in the epithelial cells, increased expression can also be seen in atherosclerotic lesions, psoriatic plaques and in human kidneys with glomerulonephritis. FKN cleavage occurs in response to inflammatory stimuli such as hypertension and diabetes, amongst other cardiovascular diseases. ADAM17 has been found to be responsible for the inducible cleavage of FKN. When FKN was transfected into host cells, inducible cleavage was blocked using the ADAM17 inhibitor TAPI-2 [57].

In human studies, plasma FKN levels were significantly higher in type 2 diabetes (T2D) patients when compared with non-diabetics and was found to correlate positively with many pro-inflammatory cytokines, including TNF-α [58]. It has been shown in human studies that those individuals with the highest FKN levels also have the highest BMI, Waist Circumference, Weight/Hip Ratio, % Fat, Blood Glucose, Insulin, HOMA-IR, Triglycerides and Total Cholesterol, as well as lowered HDL-c levels [59]. In murine studies [60], male C57BL/6 Cx3cr1^−/−^ (FKN knockout) mice display improved glucose tolerance compared with WT mice, independent of obesity. The Cx3cr1^−/−^ mice also possessed improved insulin sensitivity compared with the WT mice [60].

### IL-1R

Interleukin 1 (IL-1α and IL-1β) are major proinflammatory cytokines which have metabolic consequences. IL-1β has been shown to promote β-cell destruction in Type 1 Diabetes [61] and increase insulin resistance. IL-1α has been shown to reduce insulin signalling, as well as increase plasma triglyceride levels [62]. In addition, IL-1β plays a role in T2D [63], by its involvement in the pathogenesis of insulin resistance and ultimately promoting islet cell death. The release of IL-1β from β-cells under metabolic stress and autocrine signalling via IL1R leads to NF-κB activation and subsequent synthesis and release of IL-1β and chemokines from β-cells. The latter promotes pancreatic immune cell infiltration and cytokine release from β-cells.

Interleukin 1 ligands also play an essential role in the regulation of innate immunity and they bind to two different receptors, IL-1R1 and IL-1R2. IL-1R1 is capable of transducing cellular signals due to its cytoplasmic domain, but IL-1R2 acts as a decoy receptor for IL-1 as it lacks this cytoplasmic domain [64]. ADAM17 indirectly enhances IL-1 signalling in cells by selectively cleaving the decoy receptor IL-1R2 and in turn promotes IL-1 binding the IL-1R1 which allows signalling [64]. By changing the balance between IL-1R1 and its decoy receptor IL-1R2, ADAM17 enhances sensitivity to IL-1.

## Cofactors of ADAM17

### Rhomboid protein

The Rhomboid Proteins (iRhoms), particularly iRhom 1 and 2 are a necessary component of ADAM17 biology. IRhom is a protease which is necessary for the maturation and trafficking of ADAM17 from the endoplasmic reticulum (ER) through the Golgi and absence of cellular iRhom will result in impaired exit of ADAM17 out of the ER. [65] The iRhoms play a number of different roles including intercellular signalling, mitochondrial dynamics, parasite invasion and protein quality control [66]. Both iRhom 1 and 2 are jointly responsible for all ADAM17 activity. Beyond this, iRhom 2 can actually control the substrate specificity of ADAM17 [66].

The iRhom 2 protein has been found to be increased in obese mice with adipose tissue inflammation. When iRhom 2 is knocked out, those mice fed upon HFD had a mitigation of obesity, insulin resistance and chronic adipose tissue inflammation in comparison with that in mice with iRhom 2 overexpression [67]. With metabolic disorder there is an up-regulation of iRhom2 in the macrophages which produces TNF-α production. The iRhom 2 in macrophages facilitates the trafficking of ADAM17 and thereby promotes inflammation [67].

## ADAM17 inhibitors

### Tissue inhibitor of metalloproteinase 3: the endogenous ADAM17 inhibitor

Tissue Inhibitor of Metalloproteinase 3 (TIMP-3) is the only known endogenous inhibitor of ADAM17. It has been found to control cytokine and growth factor bioavailability so as to regulate inflammation, cell death and survival in the liver [31]. While down-regulation of TIMP-3 increases ADAM17 activity, up-regulation of TIMP-3 conversely inhibits ADAM17 activity. TIMP-3 deficient mice have also been shown to possess a heightened level of inflammation and impaired glucose tolerance due to increased levels of TNF-α caused by uncontrolled shedding [31]. The inhibitor, TIMP-3 was found to be down-regulated in adipose tissue/obesity and this correlated with an increase in ADAM17 [31]. When coupled with insulin resistance, TIMP-3 down-regulation has also been found to accelerate liver inflammation and steatosis [31].

### Exogenous ADAM17 inhibitors

ADAM17 has been found to be a promising therapeutic target for cancers of many tissues including breast, brain, colon, kidney, lung, liver, ovaries, pancreas and prostate. Thus far, exogenous ADAM17 inhibitors have been trialled in the setting of cancer.

#### Anti-ADAM17 antibody D1/GW280264X

ADAM17 is highly expressed in ovarian cancer cells. When ADAM17 was inhibited in ovarian cancer cell lines using either anti-ADAM17 antibody D1 or GW280264X, the cancer cells were sensitised to cisplatin-induced apoptosis, therefore significantly reducing cell viability [68].

#### TMI-005 (apratastat)

It has been found that ADAM17 can promote radiotherapy resistance in non-small-cell lung cancers. In *in vitro* studies, treating lung adenocarcinoma A549 cells with the ADAM17 inhibitor TMI-005, it was found that the inhibitor sensitised the tumorigenic cells to the radiotherapy. In murine studies, dual therapy with TMI-005 and radiotherapy prolonged survival in mice [69].

#### ZLDI-8

ZLDI-8 is one of the ADAM17 inhibitors that has been used to suppress the metastasis of Hepatocellular Carcinoma (HCC). It has been demonstrated that ZLDI-8 enhances the chemotherapeutic effects on tumor cell proliferation blockade, induction of apoptosis and cell cycle arrest by inhibiting the notch pathway and blocking chemical resistance [70].

#### TNF484

Aside from ZLDI-8, TNF484 is another ADAM17 inhibitor that has been shown to inhibit cell proliferation, migration and invasion of some HCC cell lines [71].

Although these exogenous ADAM17 inhibitors have been used mostly in the setting of cancer, it would be intriguing to assess their action in the setting of obesity and diabetes in animal models in the future.

### Side effects of ADAM17 inhibitors

In human studies [72], the administration of ADAM17 inhibitors in the clinical setting has proven to effectively decrease inflammatory mediators without any known side effects. In other human studies, the inhibitor INCB7839, which was used to treat breast cancer, was discontinued due to it causing an increase in deep vein thrombosis in a number of patients [73]. There have also been side effects noted after using ADAM17 inhibitors, such as musculoskeletal and liver toxicity [73]. Further studies need to be conducted to discover other novel potential side effects.

## Novel hypothesis: future research to unravel the role of ADAM17 in hyperglycaemia

Sodium Glucose Co-transporter 2 (SGLT2) helps to reabsorb up to 95% of glucose in the S1 and S2 segments of the proximal tubule. We hypothesise that in the setting of diabetes, an increase in ADAM17 elevates the renal sympathetic nervous system activity and in turn SGLT2 expression which promotes glucose reabsorption and hyperglycaemia. Glucose is an upstream mediator of hyperactivation of the sympathetic nervous system, which prevails in obesity and T2D [74]. It is interesting to note that glucose is also a stimulus for promoting expression of ADAM17 which contributes to the metabolic syndrome [55,75].

SGLT2 inhibition decreases glucose reabsorption, increases glucose excretion and is approved for use as a mode of treatment for diabetes [76]. The use of SGLT2 inhibitors has been shown to significantly reduce cardiovascular mortality and cardiovascular events [76]. Our team has shown that SGLT2 inhibition promotes sympathoinhibition and this may be a mechanism underlying cardiorenal benefits [77].

We anticipate that ADAM17 expression and activity may be reduced with SGLT2 inhibition which may decrease both sympathetic nervous system hyperactivation and hyperglycaemia.

We and others have conducted SGLT2 inhibition in mice [77]. We believe that SGLT2 inhibition should be conducted in diabetic mice and ADAM17 expression and activity should be further studied in this animal model.

## Other ADAM family members and their involvement in the metabolic syndrome

Our group has previously sought to ascertain whether the metalloproteinases ADAM19 and ADAM28 correlate with parameters of the metabolic syndrome in mice and humans. We showed for the first time in humans that both ADAM19 and ADAM28 are strongly correlated with parameters of the metabolic syndrome, particularly BMI, relative fat and the index of insulin resistance (HOMA-IR) [78,79]. We also demonstrated in our diet-induced obesity mouse model that neutralising ADAM19 therapy results in weight loss and improves insulin sensitivity [78]. In addition, down-regulation of ADAM28 with siRNA technology resulted in a lack of weight gain, promotion of insulin sensitivity/glucose tolerance, decreased liver TNF-α levels and reduced blood urea nitrogen, alkaline phosphatase and aspartate aminotransferase in our diet-induced obesity mouse model. ADAM28 knockout mice also displayed reduced body weight, elevated HDL cholesterol levels and reduction in blood urea nitrogen, alkaline phosphatase and aspartate aminotransferase [80]. Therefore, neutralisation of ADAM19 and ADAM28 may be a potential therapeutic approach to treat obesity and T2D. Clinical trials should be conducted in humans using ADAM19 and ADAM28 inhibitors.

## Conclusion

After considering our previous metabolic studies with regards to ADAM19 and ADAM28 and the fact that ADAM17 plays a multitude of roles in the pathogenesis of many diseases, it is vital to further understand the role ADAM17 plays in promoting features of the metabolic syndrome. Such studies will demonstrate that ADAM17 is a valuable therapeutic target to treat obesity and diabetes. It is highly likely that ADAM17, ADAM19 and ADAM28 work in concert to promote the metabolic syndrome.

## Open Access

Open access for this article was enabled by the participation of University of Western Australia in an all-inclusive *Read & Publish* pilot with Portland Press and the Biochemical Society under a transformative agreement with CAUL.

## Abbreviations

ACE2: angiotensin-converting enzyme type 2
ADAM17: a disintegrin and metalloproteinase 17
BMI: body mass index
FKN: Fractalkine
HCC: hepatocellular carcinoma
HDL: high-density lipoprotein
HFD: high-fat diet
IL-6: interleukin 6
iRhom: Rhomboid protein
Pref-1: pre-adipocyte factor 1
SGLT2: sodium glucose co-transporter 2
sIL-6R: soluble IL-6R
TIMP-3: tissue inhibitor of metalloproteinase 3
TNF-α: tumour necrosis factor α
T2D: type 2 diabetes
WHO: World Health Organization
